# The relationship between the leptin/ghrelin ratio and meals with various macronutrient contents in men with different nutritional status: a randomized crossover study

**DOI:** 10.1186/s12937-018-0427-x

**Published:** 2018-12-28

**Authors:** Edyta Adamska-Patruno, Lucyna Ostrowska, Joanna Goscik, Barbara Pietraszewska, Adam Kretowski, Maria Gorska

**Affiliations:** 1 Clinical Research Centre, Medical University of Bialystok, Sklodowskiej 24A, 15-276, Bialystok, MC Poland; 2 Department of Dietetics and Clinical Nutrition, Medical University of Bialystok, Mieszka I-go 4B, 15-054, Bialystok, Poland; 3 Department of Endocrinology, Diabetology and Internal Medicine, Medical University of Bialystok, Sklodowskiej 24A, 15-276, Bialystok, MC Poland

**Keywords:** Leptin/ghrelin ratio, Postprandial state, High-carbohydrate meal, High-fat meal, Normo-carbohydrate meal, Normal body weight men, Overweight/obesity

## Abstract

**Background:**

Hormones, which influence satiety and hunger, play a significant role in body energy balance regulation. Ghrelin is a peptide that plays an important role in short-term appetite regulation, whereas leptin is a factor that controls long-term energy balance and is considered as a satiety hormone. The aim of this study was to evaluate the leptin/ghrelin ratio in a fasting state and after the intake of meals with varying macronutrient contents and to assess the possible differences between normal body weight and overweight/obese men.

**Methods:**

We examined 46 healthy adult men (23 with normal body weight and 23 overweight/obese) aged 21–58, who were divided into two groups. In the crossover study, participants received isocaloric (450 kcal) meals with different macronutrient contents: men from the first group received high-carbohydrate (HC) and normo-carbohydrate (NC) meals, and in the second group, participants received high-carbohydrate and high-fat (HF) meals. The ratio of leptin/ghrelin levels was calculated from leptin and total ghrelin serum concentrations in a fasting state and 30, 60, 120, 180 and 240 min after meal intake. One-way ANOVA and Wilcoxon signed-rank tests were carried out. The normality of the variable distribution was checked with the Shapiro–Wilk test, the homogeneity of variances was verified with the Levene test, and the false discovery rate *p*-value adjustment method was used.

**Results:**

The leptin/ghrelin ratio was significantly higher in overweight/obese men than individuals with normal body weight in a fasting state, as well as postprandially. We observed trends towards a higher leptin/ghrelin ratio values from the 60 min after HC-meal intake compared to the NC- and HF-meals in normal body weight participants, while in overweight/obese men, we did not note any significant differences dependent on the meal type.

**Conclusions:**

We have observed a significantly different postprandial leptin/ghrelin ratio in normal body weight and overweight/obese men, and our results suggest that in men with normal body weight, a greater feeling of satiety may occur after high-carbohydrate meal intake, which was not noted in the overweight/obese individuals.

## Background

Obesity is a chronic disease caused mostly by an excessive supply of energy delivered with food in relation to energy expenditure, which leads to fat accumulation in adipose tissue [1]. Although the basic function of adipose tissue is energy storage, it is also an organ of endocrine secretion, which produces hormones, adipokines and cytokines [2], such as leptin, adiponectin, which are involved in body energy homeostasis and many other pathways [3]. In the central regulation of energy balance, other peripherally produced signals also participate in this process, such as fatty acids, insulin, glucagon-like peptide-1 (GLP-1), ghrelin, cholecystokinin (CCK), peptide YY (PYY) [4, 5], as well as several other molecules recently linked to energy homeostasis regulation, such as chemerin, total bile acids, fibroblast growth factor 21 (FGF-21), secreted frizzled-related protein-4 (SFRP4), irisin, and heme oxygenase-1(HO-1) [6], which can be regulated by the macronutrient composition of the diet [4–6]. As listed above, many factors are involved in energy balance control, but one of the key roles in hormonal regulation of food intake is played by ghrelin and leptin, and their interactions, since these hormones affect the energy balance antagonistically [7–9].

Ghrelin is an orexigenic hormone secreted primarily by P/D1 cells, located in the mucosa of the stomach fundus, pancreatic epsilon cells, kidneys, gonads and adipose tissue [7]. Ghrelin plays an important role in short-term appetite regulation and is characterized by increased concentrations before meal intake, and decreased levels after meal ingestion [10]. The orexigenic action of ghrelin is based on increasing gastrointestinal peristalsis and reducing insulin secretion, and despite its appetite stimulation effects, it was found that obesity, type 2 diabetes mellitus and metabolic syndrome are associated with lower serum ghrelin concentrations [7, 8, 11].

Leptin is another important signal that influences energy balance, suggested to reduce food intake and increases energy expenditure. Leptin is a hormone synthesized mainly by adipocytes but also by the stomach [12], and it inhibits appetite by counteracting the action of neuropeptide Y (NPY) and anandamines, as well as by stimulating α-Melanocyte-stimulating hormone (α-MSH) synthesis [7]. Leptin secretion is proportional to the total amount of adipose tissue, and its serum concentration increases significantly in obesity [9]. A few years ago, it was thought that leptin played a significant role only in long-term energy balance; however, the data suggest that leptin is also involved in short-term food intake and body weight regulation [10, 12]. Therefore, in the analysis of hormonal response after meal intake, it is reasonable to combine these two hormones together, and due to their interaction, the ratio of their concentrations (leptin/ghrelin ratio) is defined as a hunger signal, called the “ghrelin-leptin tango” [13]. It has been found that leptin and ghrelin concentrations depend on body weight and postprandially, on meal composition, but studies on their ratio are very limited with varying results [11, 14, 15].

Because obesity has become a global problem, the mechanisms of its development, and the identification of effective prevention and treatment strategies are of high priority. The aim of our study was to investigate the leptin/ghrelin ratio in response to meal intake with various macronutrient contents in a crossover designed study, as well as to assess the fasting and postprandial differences between normal body weight and overweight/obese men.

## Methods

The study protocol was approved by the local Ethics Committee (R-I-002/35/2009 approval of Bioethical Committee of Medical University of Bialystok, Poland). Participants were informed about the purpose and study signed informed consent consciously and voluntarily.

**Fig. 1:**
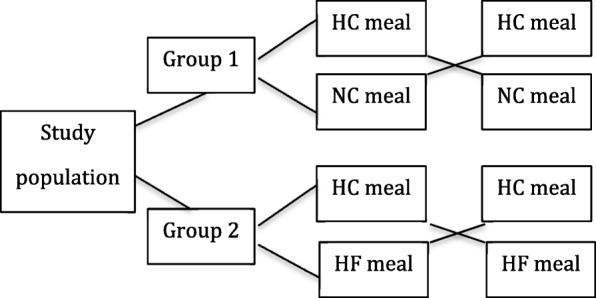
Study design. HC-meal (high-carbohydrate meal); NC-meal (normo-carbohydrate meal); HF-meal (high-fat meal)

Study design and population. The study groups, study procedures and statistical analysis were previously described in detail [16, 17], and the presented results are part of a bigger project [18–20]. Briefly, the study was conducted among 46 non-diabetic men. Anthropometric measurements (body weight, height, and calculated BMI), and percentage of adipose tissue (bioimpedance method, InBody, InBody 220, Biospace, Korea) were performed. The participants were randomly divided into two groups. Each group included men with normal body weight (BMI < 25 kg/m^2^) and overweight/obesity (BMI > 25 kg/m^2^) (Table 1). The study was carried out by the crossover method. The subjects received standardized isocaloric (450 kcal) meals with different contents of basic nutrients during two subsequent visits (Fig. 1). Group I: 11 men with normal body weight (N1) and 12 subjects with overweight/obesity (O/O1) received a high-carbohydrate/fat-free meal (HC-meal, *Nutridrink Fat Free, Nutricia, Poland*) and a normo-carbohydrate/high-protein meal (NC-meal, *Cubitan, Nutricia, Poland*); and Group II: 12 men with normal body weight (N2), and 11 subjects with overweight/obesity(O/O2) received the high-carbohydrate/fat-free meal (HC-meal, *Nutridrink Fat Free, Nutricia, Poland*) and a high-fat/low-carbohydrate meal (HF-meal, *Calogen, Nutricia, Poland*). The energy and macronutrient contents for all meals are presented in Table 2. The study duration was approximately 3 weeks and included a screening visit and two meal challenge test visits, during which participants received the test meals, with 1–2 week intervals between tests. Subjects were asked to maintain their usual diet and lifestyle throughout the study. After at least 12-h fasting, at approximately 08.00–08.30 AM, all anthropometric measurements were performed, and a cannula was inserted into the vein. To determine leptin and ghrelin concentrations, which were used for leptin/ghrelin ratio calculations, venous blood was collected immediately prior to meal intake (0 min), and at 30, 60, 120, 180 and 240 min after meal intake.

**Table 1:** Study population characteristics

		Normal body weight men	Overweight/obese men	*p*-value
Group I	n	11	12	
	Age (years)	33 ± 2	40 ± 2	0.01
	BMI (kg/m^2^)	23.8 ± 0.5	31.4 ± 1.5	0.0002
	Body fat content (%)	17.9 ± 1.0	28.6 ± 1.7	0.00003
Group II	n	12	11	
	Age (years)	33 ± 3	36 ± 3	0.24
	BMI (kg/m^2^)	23.9 ± 0.2	33.7 ± 2.2	0.000001
	Body fat content (%)	18.6 ± 1.5	31.9 ± 2.7	0.0002

The results are presented as mean values ± SE

**Table 2:** The energy and macronutrient composition of tested meals

	High-carbohydrate meal	Normo-carbohydrate meal	High-fat meal
Energy (kcal)	450	450	450
Carbohydrate (g)	100.5	51.1	4.0
Carbohydrate (% of total energy)	89.3	45.1	4.0
Fat (g)	0	12.6	47.5
Fat (% of total energy)	0	25.2	96
Protein (g)	12	36	0
Protein (% of total energy)	10.7	29.7	0
Fibre (g)	0	0.1	0

Biochemical analysis. Blood was drawn, centrifuged, stored at − 80 °C, and prepared for testing as previously described, in accordance with the recommendations and procedures provided by the laboratory kit producers. Leptin concentration was measured using an enzyme immunoassay (Human Leptin ELISA, BioVendor, Czech Republic). Total ghrelin concentration was measured using a radioimmunoassay (Ghrelin (total) RIA, Millipore, USA).

Statistical analysis. The statistical analysis has been described in detail previously [17]. The mean value and its standard error were calculated, and two hypotheses were stated: (1) the type of meal does not influence the postprandial leptin/ghrelin ratio (the normal body weight and overweight/obese men were analysed separately), and (2) there is no statistically significant difference in postprandial leptin/ghrelin ratio between normal body weight and overweight/obese individuals (the types of meals were analysed separately).

Since participants in every group received two different meals, the lack of independence was taken into consideration. One-way ANOVA (analysis of variance), or the Wilcoxon signed-rank test, both for paired samples, were carried out, dependent on fulfilment of the normality of variable distribution, which was checked by the Shapiro–Wilk test.

The goal of the second hypothesis was to investigate whether there are any significant differences in the postprandial leptin/ghrelin ratio between normal body weight and overweight/obese men. We used one-way ANOVA or the Wilcoxon rank-sum test, depending on the normality of variable distribution, and the homogeneity of variances (verified with the Levene test). The false discovery rate *p*-value adjustment method was used to address the issue of multiple hypothesis testing. For all analyses, the alpha level was set at 0.05.

## Results

In Group I, the leptin/ghrelin ratio after the HC-meal intake vs. the leptin/ghrelin ratio after the NC-meal intake was analysed. In N1 subjects we observed a trend towards higher leptin/ghrelin ratio values from 60 min after the HC-meal compared to the NC-meal (Table 3). In O/O1 men we did not notice any significant differences in the leptin/ghrelin ratio between both meals.

**Table 3:** Comparison of the leptin/ghrelin ratio after the HC- and NC-meal intake, in the population of study Group I

G1	Time (min)	0	30	60	120	180	240
High-carbohydrate (HC) meal vs normo-carbohydrate (NC) meal	N1-HC	5.01 ±1.75	4.71 ±1.55	5.28 ±2.00	6.74 ±2.39	5.59 ±1.79	4.92 ±1.47
	N1-NC	3.50 ±1.21	4.25 ±1.63	3.11 ±0.80	3.04 ±0.96	3.32 ±0.91	2.92 ±0.67
	†p=	0.59	0.57	0.17	0.06	0.07	0.14
	O/O1-HC	21.00 ±5.25	17.44 ±4.91	17.84 ±3.74	18.40 ±3.72	20.05 ±4.41	17.63 ±4.36
	O/O1-NC	19.25 ±7.10	16.49 ±4.05	19.87 ±4.33	24.15 ±8.84	20.85 ±5.28	19.15 ±4.40
	‡p=	0.26	0.57	0.67	1.0	0.34	0.68

The results are presented as mean values ± SE. † comparison between the high-carbohydrate (HC) and normo-carbohydrate (NC) meals in the normal body weight (N1) group; ‡ comparison between the high-carbohydrate (HC) and normo-carbohydrate (NC) meals in the overweight/obesity (O/O1) group

In Group II, the leptin/ghrelin ratio after the HC-meal intake vs. the leptin/ghrelin ratio after the HF-meal intake was analysed. In N2 subjects we noted a trend towards higher leptin/ghrelin ratio values at 60 min of testing after the HC-meal intake when compared to the HF-meal, and at 120 min the difference almost reached statistical significance (*p* = 0.06), which was not observed in the O/O2 subjects (Table 4).

**Table 4:** Comparison of the leptin/ghrelin ratio after the HC- and HF-meal intake, in the population of study Group II

G2	Time (min)	0	30	60	120	180	240
High-carbohydrate (HC) meal vs high-fat (HF) meal	N2-HC	7.02 ±1.49	7.43 ±1.41	8.70 ±1.73	8.83 ±2.10	8.18 ±1.68	7.31 ±1.25
	N2-HF	7.01 ±1.66	6.19 ±1.45	6.97 ±1.77	6.91 ±1.54	7.55 ±2.06	6.84 ±1.51
	†p=	0.99	0.22	0.10	0.06	0.63	0.67
	O/O2-HC	38.21 ±14.50	31.55 ±12.12	30.58 ±10.20	32.45 ±10.82	37.57 ±14.44	28.04 ±10.01
	O/O2-HF	27.50 ±10.84	29.77 ±10.11	29.76 ±9.51	33.56 ±11.89	25.63 ±7.84	28.37 ±8.38
	‡p=	0.27	0.88	0.59	0.45	0.22	0.50

The results are presented as mean values ± SE. † comparison between the high-carbohydrate (HC) and high-fat (HF) meals in the normal body weight (N2) group; ‡ comparison between the high-carbohydrate (HC) and high-fat (HF) meals in the overweight/obesity (O/O2) group

**Fig. 2:**
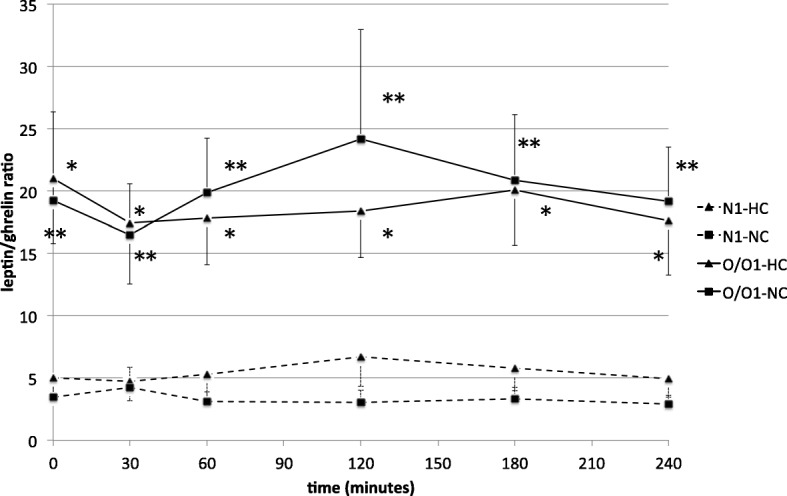
Comparison of the leptin/ghrelin ratio between normal body weight and overweight/obese men. The results are presented as mean values ± SE. N1, broken line- men with normal body weight; O/O1, solid line- overweight/obese subjects; HC- high-carbohydrate meal; NC- normo-carbohydrate meal. The comparison between the N1 and O/O1 study groups after HC-meal intake *p < 0.05, and after NC-meal intake **p < 0.05

**Fig. 3:**
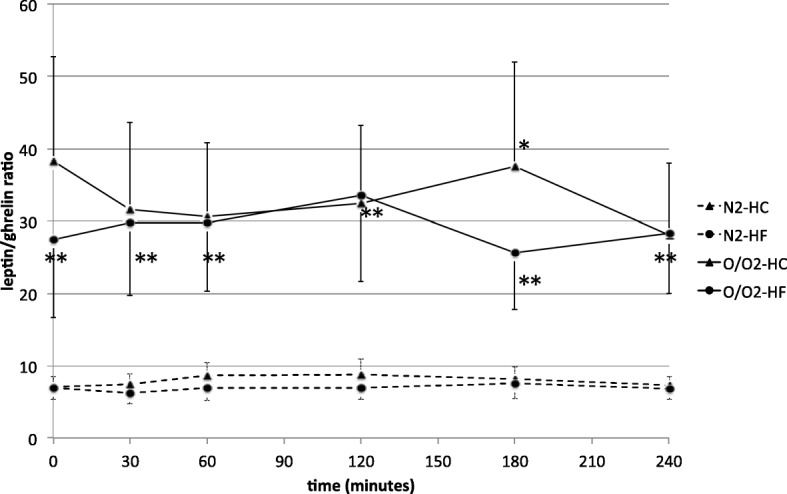
Comparison of the leptin/ghrelin ratio between normal body weight and overweight/obese men. The results are presented as mean values ± SE. N2, broken line- men with normal body weight; O/O2, solid line- overweight/obese subjects; HC- high-carbohydrate meal; HF- high-fat meal. The comparison between the N2 and O/O2 study groups after HC-meal intake *p < 0.05, and after HF-meal intake **p < 0.05

Subsequently, the leptin/ghrelin ratio was compared between normal body weight and overweight/obese individuals after meal intake in Group I (Fig. 2) and Group II (Fig. 3). We noticed that the leptin/ghrelin ratio was significantly higher in overweight/obese men, compared to normal body weight participants in a fasting state, as well as postprandially in both groups and after all the three meals. A more favourable leptin/ghrelin ratio in normal body weight men was observed after the HC-meal intake compared to the NC- and HF-meals, while in overweight/obese individuals, we did not note any significant differences dependent on the meal type.

## Discussion

Energy balance is regulated by central and peripheral signals, but the final clinical effect is not a consequence of the individual action of only one hormone, but depends on their interactions [9]. Therefore in our study we analysed the ratio of two crucial hormones that impact hunger and satiety feelings- the leptin to total ghrelin concentrations. The main finding of our study is that in normal body weight men, a more beneficial leptin/ghrelin ratio was noted after the HC-meal intake, compared to the NC- and HF-meals. The other observation is that overweight/obese men presented with a significantly higher leptin/ghrelin ratio in a fasting state, as well as after intake of each of the three meals, compared to normal body weight participants.

Our results seem to confirm the observations of Sanchez J et al. [21], who noted higher inhibition of stomach ghrelin production after a high-carbohydrate diet compared to a high-fat diet in Wistar rats. Moreover, the authors also noticed that carbohydrate intake (but not fat intake) stimulated gastric leptin expression, though the gastric leptin levels decreased after food intake without any differences between carbohydrate or fat intake. Other authors [22] noted that a higher leptin to ghrelin ratio was significantly correlated with a lower resting metabolic rate. These results are consistent with the findings of our studies, since in the overweight/obese group, in which we observed a higher leptin/ghrelin ratio in the current study, we also noted in a previously published analysis a lower energy expenditure in a fasting state, as well as postprandially [17]. It has been shown that leptin increases energy expenditure in particular through its effects on the cardiovascular system, as well as on brown adipose tissue thermogenesis via the hypothalamus [23], while ghrelin was found to decrease energy expenditure, mainly through stimulating the activity of neuropeptide Y/agouti-related protein (NPY/AgRP) and hypocretin/orexin cells, and decreasing the activity of proopiomelanocortin (POMC) and corticotropin releasing hormone (CRH) cells [24].

A significantly higher leptin/ghrelin ratio was observed in overweight/obese subjects in a fasting state, as well as after intake of each of the tested meals, which undoubtedly is a result of its higher fasting level in overweight/obese men. However, we have noted that in normal body weight men, there is strong tendency for an increased leptin/ghrelin ratio after the HC-meal than after the NC-meal intake, especially after 120–180 min. The opposite trends were noted in the overweight/obese group, where the increase of the leptin/ghrelin ratio was more marked after the NC-meal intake, while after the HC-meal, the mean values decreased postprandially. However, the differences were not significant, most likely due to the high standard error. We observed similar results in the G2 group. The tendency of an increase the leptin/ghrelin ratio was noted in normal body weight men after the HC- compared to the HF-meal, especially 120 min after meal intake, while in the overweight/obese group, the mean values decreased postprandially, and increased after the HF-meal intake, but the differences were not significant, maybe due to too high standard error, or too small a study sample.

To our knowledge, there are not any data that we can compare our leptin/ghrelin ratio results to, but different ghrelin and leptin concentrations have been demonstrated to be dependent on nutritional status [11], as well as on meal composition [25]. We decided to analyse leptin and ghrelin together, as the leptin/ghrelin ratio, since these are two of the main hormones that influence the metabolic status of the body, appetite and hunger sensations, etc., which are an effect of the hormone interactions, rather than an effect of only one individual hormone’s action. Therefore, with a higher leptin/ghrelin ratio, we can expect more beneficial effects in suppressing the feeling of hunger. However neither the relationship between leptin/ghrelin ratio to hunger and satiety regulation, nor the metabolic states and consequences, have been published so far. Previous studies have found that increased ghrelin to leptin ratio correlated with increased hunger and appetite [26, 27]; therefore, we can suppose that with a higher leptin to ghrelin ratio, hunger and appetite should decrease. The only three studies that analysed the leptin/ghrelin ratio were focused on the relationship with child obesity [28] and resting metabolic rate, and this ratio was considered as a possible biomarker for predicting metabolic adaptations to energy restriction treatment [22], and as a non-invasive tool for the discrimination of patients with obesity who are more likely to regain weight after therapy for obesity [29].

Our experiment also has some limitations. The presented study is a part of our larger project, with very long and labourious protocol procedures, and it was difficult to find volunteers who agreed to participate in the three meal challenge tests, with all of the tested meals. Therefore, if we wanted to follow a crossover study design, it was needed to divide participants into two groups (Group I and Group II) to compare the effects of different meals intake in the same individuals. The other major limitation is that normal body weight men in Group 1 were a little bit younger than the overweight/obese individuals, what could affect the results when comparing the differences after the HC- and NC-meal intake dependently on the body weight in this group, but it did not affect the results from comparing the HC- with NC-meal. The other limitations include the small sample size, enrolling only the male participants, and the liquid form of meals. Some of them were intentional, to reduce the impact of possible confounding factors, such as the influences of sex hormones and sex differences; or to decrease the time of meal digestion and absorption, to not discourage the volunteers with a long time spent at each visit etc. However, the mentioned factors could affect our results, and therefore, our observations and the relationship between leptin/ghrelin ratio and metabolic changes need further investigation.

## Conclusions

In conclusion, the trends observed in our study show that in men with normal body weight, we can expect a more beneficial leptin/ghrelin ratio after HC-meal intake, whereas in overweight/obese individuals, we can expect this after meals with limited carbohydrate content. Therefore, the recommendation for overweight/obese men to choose meals with lower carbohydrate content, could be a practical implication of this study.

## Abbreviations

AgRP: Agouti-related protein
ANOVA: One-way analysis of variance
BMI: Body mass index
CCK: Cholecystokinin
CRH: Corticotropin releasing hormone
ELISA: Enzyme-linked immunosorbent assay
FGF-21: Fibroblast growth factor 21
GLP-1: Glucagon-like peptide-1
HC: High-carbohydrate
HF: High-fat
HO-1: Heme oxygenase-1
NC: Normo-carbohydrate
NPY: Neuropeptide Y
NPY: Neuropeptide Y
POMC: Proopiomelanocortin
PYY: Peptide YY
RIA: Radioimmunoassay
SFRP4: Secreted frizzle-related protein-4
α-MSH: Stimulating the α-Melanocyte-stimulating hormone
α-MSH: α-Melanocyte-stimulating hormone
